# Maghemite Intercalated Montmorillonite as New Nanofillers for Photopolymers

**DOI:** 10.3390/nano2040413

**Published:** 2012-11-19

**Authors:** Bassam Tarablsi, Christelle Delaite, Jocelyne Brendle, Celine Croutxe-Barghorn

**Affiliations:** 1 Laboratoire de Photochimie et d’Ingénierie Macromoléculaires, Ecole Nationale Supérieure de Chimie de Mulhouse, Université de Haute-Alsace, 3 rue A. Werner, 68093 Mulhouse Cedex, France; Email: bassamta27@hotmail.com (B.T.); christelle.delaite@uha.fr (C.D.); barghorn@uha.fr (C.C.-B.); 2 Equipe Matériaux à Porosité Contrôlée, Institut de Science des Matériaux de Mulhouse, LRC-CNRS 7228, Ecole Nationale Supérieure de Chimie de Mulhouse, Université de Haute-Alsace, 3 rue A. Werner, 68093 Mulhouse Cedex, France

**Keywords:** montmorillonite, ion exchange, maghemite, nanocomposites, photopolymerization

## Abstract

In this work, maghemite intercalated montmorillonite (γFe_2_O_3_-MMT)/polymer nanocomposites loaded with 1 or 2 wt.% of nanofillers were obtained by photopolymerization of difunctional acrylate monomers. The γFe_2_O_3_-MMT nanofillers were prepared by a new method based on the *in situ* formation of maghemite in the interlayer space of Fe-MMT using a three step process. X-ray diffraction (XRD), chemical analysis, TG/DTA and transmission electron microscopy (TEM) characterization of these nanofillers indicated the efficiency of the synthesis. When following the kinetics of the photopolymerization of diacrylate-γFe_2_O_3_-MMT nanocomposites using FTIR spectroscopy no significant inhibition effect of the nanofillers was observed at a loading up to 2 wt.%. These innovative nanocomposites exhibit improved mechanical properties compared to the crude polymer.

## 1. Introduction

Nanocomposites based on polymers have attracted attention due to their superior engineering properties compared to the neat polymer and to the classical composites [1,2,3,4,5]. Among them, nanocomposites containing iron oxide nanoparticles or clays have gained great interest in recent decades because of their unique properties [6,7,8,9,10,11,12]. Indeed, the magnetic properties of iron oxides such as Fe_3_O_4_ (magnetite) or γ-Fe_2_O_3_ (maghemite) impart applications in various fields such as high-density magnetic recording, pigmentation, microwave absorbing coatings, magnetic cooling, and are also suitable for medical applications (such as magnetic targeting, drug delivery, contrast enhancement in magnetic resonance imaging, and magnetic hyperthermia) [13,14,15,16,17,18]. The most widely preparative methods used for the synthesis of magnetic iron oxide nanoparticles in an aqueous suspension are based on the co-precipitation of iron salts in alkaline medium [19].

Due to their nanoscale particles size and their layered structure, swelling clays offer an attractive way to enhance mechanical, thermal and barrier properties of polymers [20]. Their structure consists of layers made up of two tetrahedrally coordinated silicon atoms fused to an edge-shared octahedral sheet of either aluminium or magnesium hydroxide. The layer thickness is around 1 nm. Stacking of the layers leads to a regular Van Der Waals gap between the layers called the interlayer. Isomorphic substitution within the layers (for example, Si^4+^ replaced by Al^3+^) generates negative charges that are counterbalanced by cations such as Na^+^ in the interlayer space. The hydrophilicity of swelling clays can be turned into hydrophobicity by exchanging the interlayer cations with organic cations rendering them compatible with organic matrices [21,22].

Whatever the filler, polymer nanocomposites are usually prepared by *in situ* polymerization (thermal polymerization or more seldom, by UV curing), sol–gel processing, or melt compounding [23,24,25,26,27,28]. Although nanocomposites containing either clays [29,30] or iron oxide nanoparticles [31,32,33] are extensively described in the literature only a few works describe the use of mixtures of both [34,35,36]. Laachachi *et al.* [34], for example prepared, by melt blending, PMMA-γFe_2_O_3_-organomodified montmorillonite nanocomposites presenting improved thermal stability and flammability properties compared to unfilled PMMA. Until now, no work has been reported on the preparation of such nanocomposites using UV-curable resins, in spite of the several advantages that this process presents over thermal polymerization. Indeed, photopolymerization leads to high polymerization rates; it is less energy consuming, occurs at room temperature, and is environmentally friendly due to the absence of VOC. Therefore it is a curing process at low cost.

Our objective in this study was to prepare a new kind of nanocomposite based on particles of maghemite and montmorillonite in a photopolymeric matrix. For this purpose, maghemite intercalated montmorillonite (γFe_2_O_3_-MMT) was prepared using (1) ion exchange of interlayer sodium ions with iron (III) ions, (2) formation of goethite and (3) thermal solid-state transformation of goethite into maghemite intercalated montmorillonite. Then, this new nanofiller was incorporated in photopolymerizable formulations containing 1,6-hexanediol diacrylate (HDDA) and polyethylene glycol (400) diacrylate (Sr 344) before UV curing. The influence of the nanofiller (γFe_2_O_3_-MMT) content on the kinetics of photopolymerization was determined and some properties of the final nanocomposites were evaluated.

## 2. Results and Discussion

### 2.1. Nanofillers Preparation (γFe_2_O_3_-MMT)

#### 2.1.1. Ion Exchange Reaction

**Figure 1:**
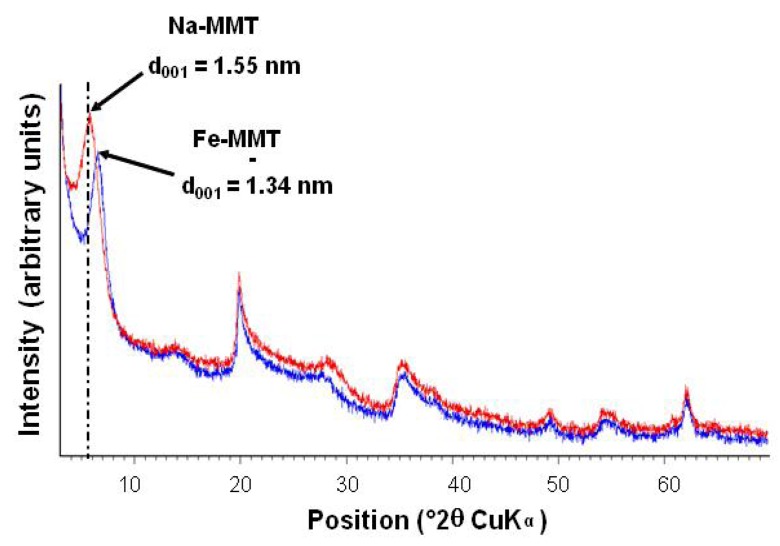
Comparison of the X-ray diffraction (XRD) patterns of Na-montmorillonite (Na-MMT) (red) and maghemite-intercalated montmorillonite (γFe_2_O_3_-MMT) (after aqueous exchange with Fe^3+^ ions).

Figure 1 displays the comparison between the X-ray diffraction (XRD) patterns of Na-MMT and Fe-MMT. It appears that the ion exchange led to a decrease of the d_001_ value (from 1.55 nm for the pristine Na-MMT to 1.34 nm for Fe-MMT) which can be attributed to the replacement of sodium cations (Na^+^ = 0.095 nm) by iron cations (Fe^3+^ = 0.064 nm). The determination of the sodium and iron contents in Fe-MMT samples by X-ray fluorescence indicated that the iron content equals 2.5 wt.% and that 97% of the initial Na cations were replaced by Fe cations. These results and the orange coloration of the sample confirmed a successful intercalation of the iron species.

#### 2.1.2. Formation of Goethite

**Figure 2:**
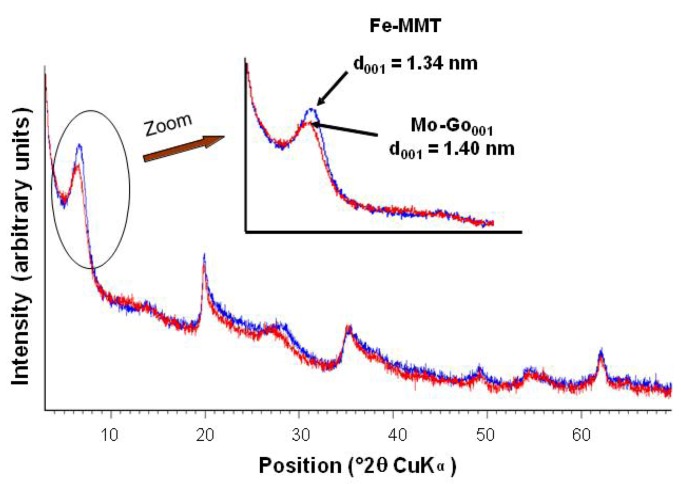
Comparison of the XRD patterns of Fe-MMT(blue) and Mo-Go_001_ (red).

Goethite particles can be formed from a ferric salt solution in two different ways using either a basic [37] or an acidic route [38]. In our case, and for the first time, goethite was formed in the interlayer space of the montmorillonite starting from the Fe cations previously introduced by ion exchange. Both routes were performed and the products obtained (Mo-Go_001 _and Mo-Go_002_) exhibited the same characteristics. The X-ray diffraction pattern of Mo-Go_001,_ given as an example in Figure 2, showed a very weak increase of the interlayer space of the montmorillonite. 

**Figure 3:**
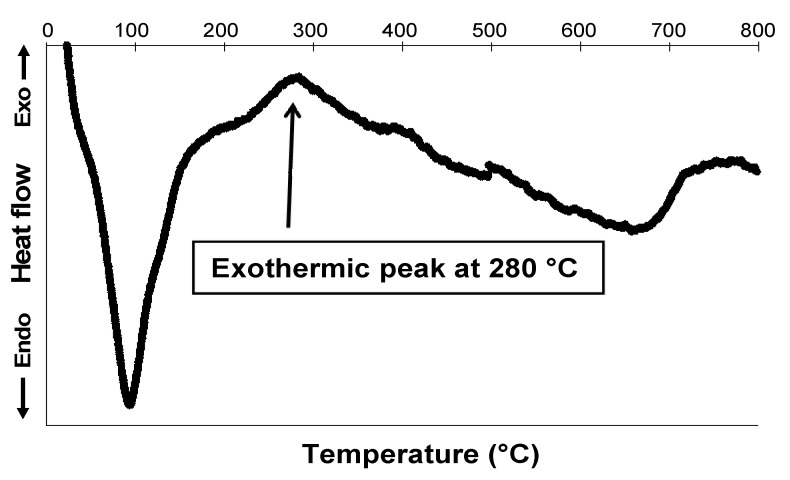
Differential Thermal Analysis (DTA) curve of Mo-Go_001_.

As expected, the iron contents (2.5 wt.%) of both products did not change after treatment. Due to the small amount of iron in the sample, it was not possible to detect goethite by conventional methods (*i.e.*, XRD). Several studies of decomposition of goethite into maghemite by heating (between 200 and 280 °C) have been reported [39,40]. The curve of heat flow versus temperature for Mo-Go_001 _is shown in Figure 3 as an example. The sample exhibits a main exothermic peak at 280 °C attributed to the transformation of goethite into maghemite, thus proving the formation of goethite.

As the two routes led to the same results the following study was pursued with the sample Mo-Go_001_.

#### 2.1.3. Thermal Solid-State Transformations

**Figure 4:**
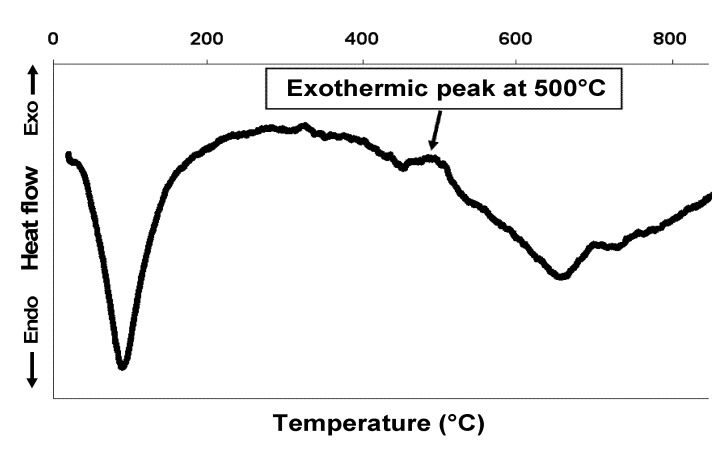
DTA curve of Mo-Mag_001_.

Thermal treatment of Mo-Go_001_ was performed in order to transform goethite into maghemite leading to a brown sample named Mo-Mag_001_. This color is characteristic of maghemite nanoparticles [40]. The curve of heat flow versus temperature of Mo-Mag_001_ is shown in Figure 4. The Differential Thermal Analysis (DTA) curve shows the disappearance of the exothermic peak at 280 °C, which proves that all of the goethite was transformed by the thermal treatment applied in the previous steps. The appearance of a new exothermic peak at around 500 °C is attributed, according to the literature, to the transformation of maghemite into hematite [6,41].

**Figure 5:**
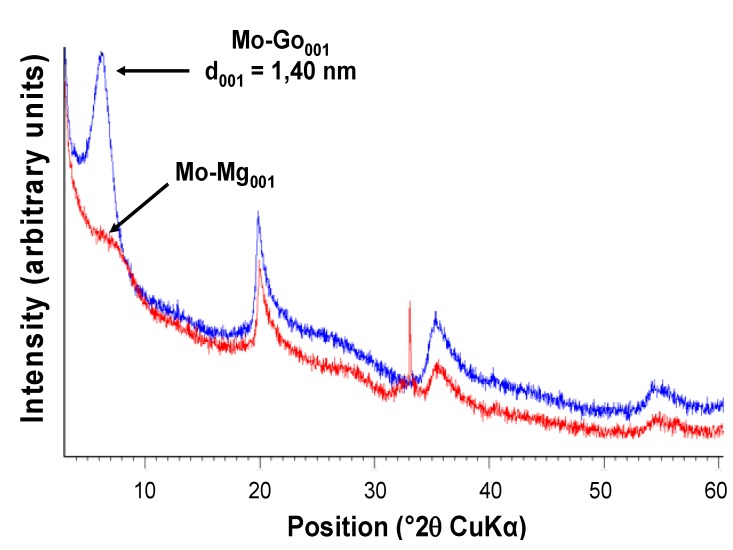
Comparison of the XRD patterns of Mo-Go_001_ (blue) and Mo-Mag_001_ (red).

**Figure 6:**
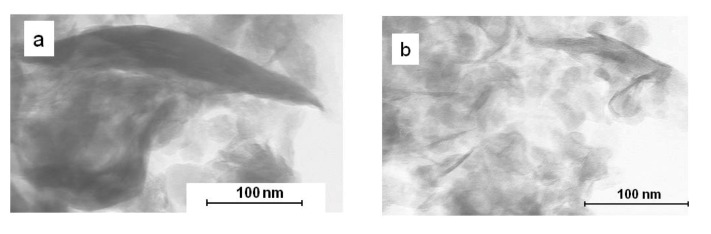
Transmission electron microscopy (TEM) micrographs of (**a**) Na-MMT and (**b**) Mo-Mag_001_.

The X-ray diffraction pattern (Figure 5) shows that the thermal treatment led to a broadening of the (001) reflexion. This result may be explained by the delamination of montmorillonite layers induced by the generation of maghemite nanoparticles in the interlayer space as a new peak, which may be attributed to maghemite, is observed on the Mo-Mag_001_ diffractogram [36]. This hypothesis is supported by TEM analysis as Mo-Mag_001 _is partially exfoliated (Figure 6b) even if no maghemite nanoparticles can be observed on the micrographs.

### 2.2. Kinetics of Photopolymerization

**Figure 7:**
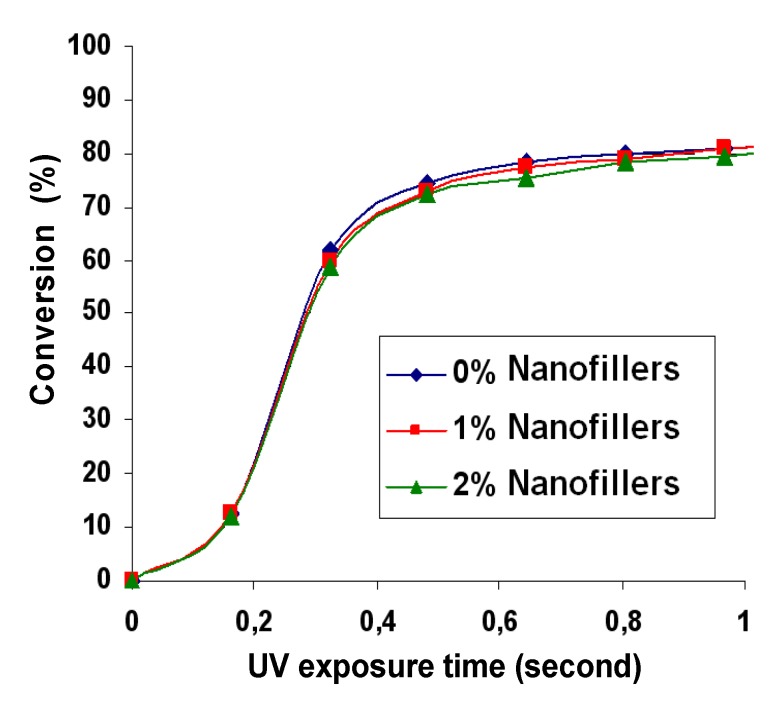
Conversion curves depending on the nanofiller content.

**Figure 8:**
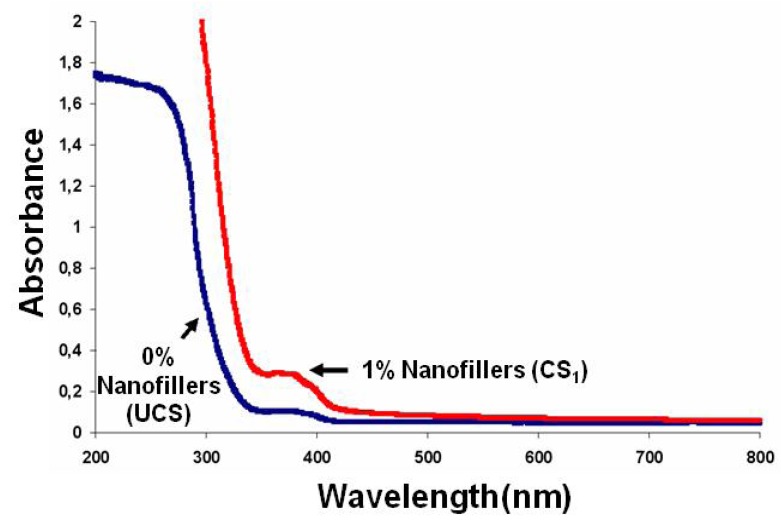
UV absorption spectra of crude polymer (UCS) and nanocomposite CS_1_.

The degree of conversion of the UV exposed samples was evaluated by infrared spectroscopy, by monitoring continuously the disappearance of the characteristic bands of the reactive group, *i.e.*, at 1590 cm^−^^1^ or 1660 cm^−^^1^ for the acrylate double bond. The addition of low concentrations of nanofillers (1 and 2 wt.%) to the resin had no negative effect (inner filter effect) on the polymerization kinetics in thin films (10 µm), as shown by the conversion versus time curves reported in Figure 7. This observation can be explained by the very weak UV absorption of the dispersed nanofillers in the absorption range of the photoinitiator (330 to 410 nm) allowing the photopolymerization process to completely cure the films (Figure 8).

### 2.3. Nanocomposite Morphology

**Figure 9:**
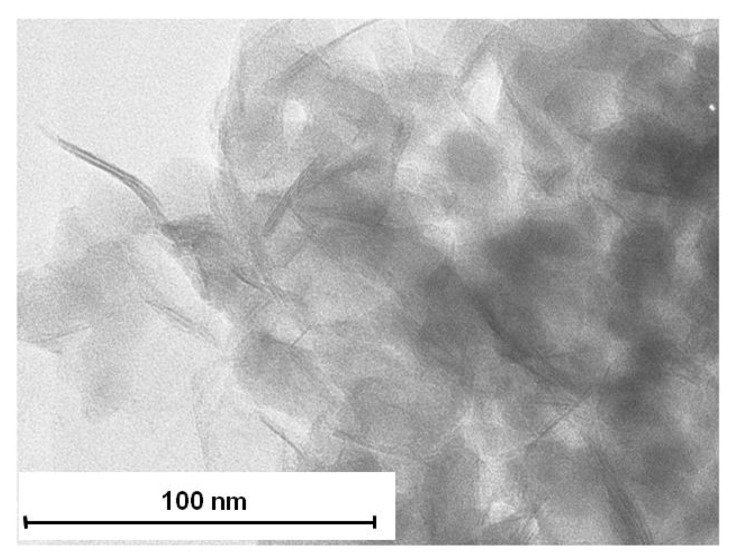
TEM micrographs of CS_1 _nanocomposite.

The TEM micrograph of the CS_1_ nanocomposite indicates that the γFe_2_O_3_-MMT filler is still partially exfoliated (Figure 9).

### 2.4. Properties of Nanocomposite Films

#### 2.4.1. Viscoelastic Properties

**Figure 10:**
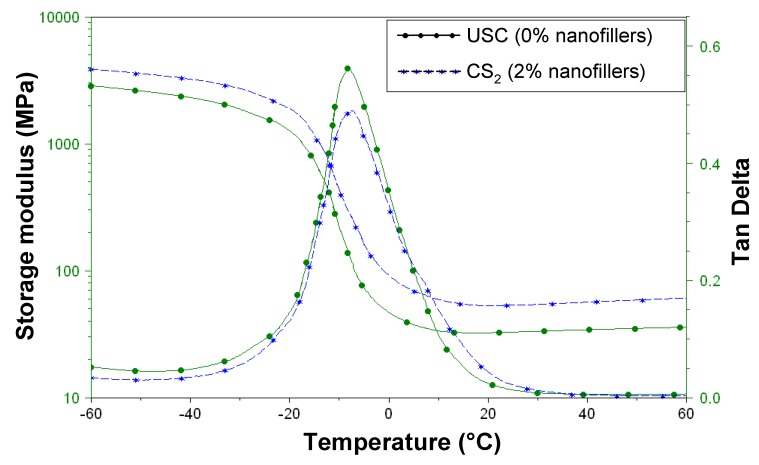
Viscoelastic properties of crude polymer (UCS) and CS_2_ nanocomposite.

The viscoelastic properties of the nanocomposites were evaluated by dynamic mechanical analysis on 100 µm thick samples and compared to the pure polymer. Figure 10 displays typical profiles for polymer and CS_2_ films by monitoring the variation of the storage modulus (E’) and the loss factor (tan δ) with increasing temperature. Youngs modulus and glass transition temperatures are gathered in Table 1.

**Table 1:** Glass transition temperatures and Youngs modulus.

Sample	Tg (°C)	Youngs modulus (MPa) at 25 °C
crude polymer (UCS)	–7.1	32
CS_2_	–7.3	53

The high storage modulus for the CS_2_ sample compared to the crude polymer (53 and 32 MPa respectively) shows a significant reinforcement effect of the filler. On the contrary, no effect was observed on the glass transition temperature.

#### 2.4.2. Hardness Properties

Table 2 shows that the addition of nanofillers in the formulation has no influence on the polymer hardness as already observed for UV cured acrylate-MMT nanocomposite [42].

**Table 2:** Hardness and gloss of the CS_1_ and CS_2_ nanocomposites depending on the filler content.

Sample	Hardness (s)	Gloss at 20° (%)
crude polymer (UCS)	195 ± 10	100
CS_1_	190 ± 10	91
CS_2_	192 ± 10	83

#### 2.4.3. Gloss Properties

Results gathered in Table 2 indicate a decrease of the gloss at 20° of the filler content which could be attributed to an increase of the surface roughness [41].

## 3. Experimental Section

### 3.1. Materials

Sodium acetate (NaCOOCH_3_, 99%; Fluka, Saint-Quentin Fallavier, France), pseudo-boehmite (Al_2_O_3_, 75%–78%, Pural SB1, Condea, Hambourg, Germany) Silica (SiO_2_, 99.5%, Aerosil 130; Degussa, Evonik, Rheinfeld, Germany), Iron (III) chloride hexahydrate (Strem Chemicals, 97%), hydrochloric acid (37%, Riedel-de Haën), sodium hydroxide (99%, Aldrich, Saint-Quentin Fallavie, France), 1,6-hexanediol diacrylate (HDDA, 80%, Aldrich, Saint-Quentin Fallavier, France), polyethylene glycol (400) diacrylate (SR 344, Aldrich, Saint-Quentin Fallavier, France), bis (2,4,6-trimethylbenzoyl)-phenylphosphine oxide (Irgacure 819, 100%, BASF, Ludwigshafen, Germany), hydrofluoric acid (HF, 40%; BDH, diluted to 5%) were used as received. The chemical formula of HDDA and SR344 are gathered in Table 3.

**Table 3:** Chemical structures of the UV-curable acrylic resins.

Symbol	Name	Structure
HDDA	1,6-Hexanediol diacrylate	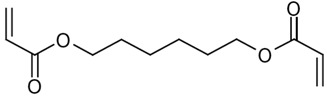
SR 344	Polyethylene glycol (400) diacrylate	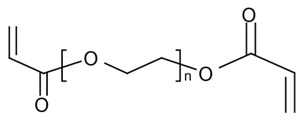

### 3.2. Procedures

#### 3.2.1. Preparation of Montmorillonite

Na-montmorillonite (Na-MMT) having the following chemical composition per half a unit cell: Na_0,11_ (Al_1,6_ Mg_0,45_) Si_4 _O_10_ (OH,F)_2_ was prepared according to Reinholdt *et al.* [44,45].

#### 3.2.2. Preparation of γFe_2_O_3_-MMT

**Figure 11:**
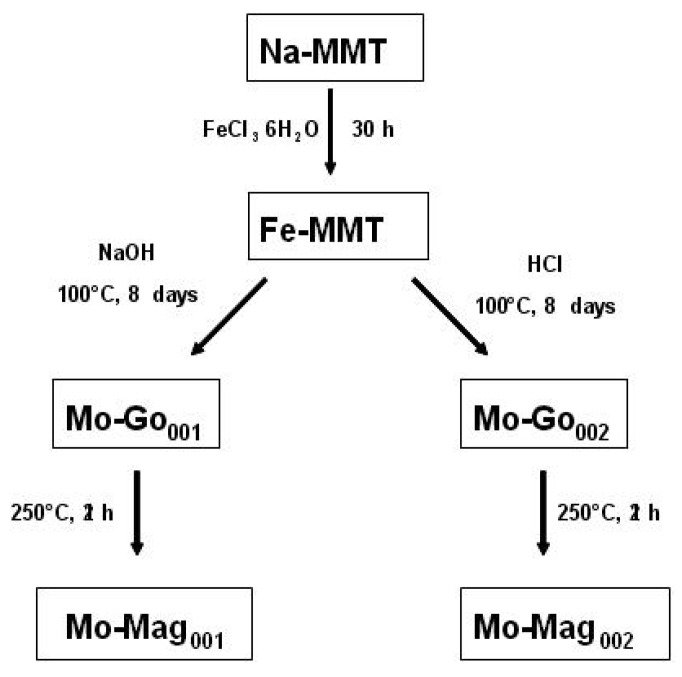
Synthetic pathways for the nanofillers Mo-Mag_001_ and Mo-Mag_002_.

Maghemite-intercalated montmorillonite (γFe_2_O_3_-MMT) was prepared by a three stage process outlined in Figure 11.

##### - Ion Exchange

For the ion exchange reaction, 1.0 mmol of FeCl_3_ was added dropwise into 20 mL of a 5 wt.% Na-MMT suspension in HCl solution (0.1 M). After 1h of sonication, the mixture was stirred at room temperature over 24 h. The solid was then recovered by centrifugation, washed thoroughly with distilled water and dried at 70 °C for 12 h. The orange colored Fe-MMT sample obtained was then ground to a fine powder.

##### - Goethite Formation

Two methods were used:

- The basic route [35]: 1 g of Fe-MMT was dispersed in 11 mL of water. Then 9.3 mL of a 5.4 M sodium hydroxide aqueous solution were added dropwise. The OH^- ^to Fe^3+^ molar ratio and the pH were respectively 5.4 and 12.3.
- The acidic route [36]: 1 g of Fe-MMT was dispersed in 23 mL of a 0.01 M hydrochloric acid aqueous solution.

In both cases, the suspensions were hydrothermally treated at 100 °C over 8 days before being cooled to room temperature, centrifuged, washed with distilled water and dried at 70 °C for 12 h and finally ground into a powder. The obtained solids were labelled Mo-Go_001_ (basic route) and Mo-Go_002_ (acidic route).

##### - Thermal Treatment

Sample Mo-Go_001 _was heated at 250 °C for 2 h under air in order to form γFe_2_O_3_-MMT brown powder, named Mo-Mag_001_.

#### 3.2.3. Preparation of the Formulations and Photopolymerization

##### - Preparation of CS_1_ and CS_2_ Formulations

A liquid UV-curable resin was first obtained by mixing 10 wt.% of HDDA (SR238) and 90 wt.% of Polyethylene glycol (400) diacrylate (SR 344). Then 3 wt.% of Irgacure 819 and 1 wt.% (CS_1_) or 2 wt.% (CS_2_) of Mo-Mag_001_ were added and the mixture was sonicated for 3 h at room temperature (Fischer scientific Sonicator S-LINE).

##### - Photopolymerization

The liquid resin (CS_1_ or CS_2_) was applied onto a BaF_2_ crystal by means of a calibrated wire-wound applicator. Then 10 µm thick films with a diameter of 20 mm were exposed to a polychromatic medium pressure Hg/Xe lamp (Hamamatsu LC5 L8222-01) equipped with a reflector at 366 nm and coupled with a flexible light-guide. The end of the optical guide was placed at a distance of 3 cm from the sample and directed at an incident angle of 90° onto the sample window. The intensity was of 104 mW/cm^2^ measured by an International Light IL-390 radiometer. The photopolymerization process was monitored *in situ* by Real-Time Fourier Transform InfraRed spectroscopy (RT-FTIR IFS 66S from Bruker Optics) by monitoring the decay of the IR absorption bands at 1590 cm^−1^ or 1660 cm^−1^ for the acrylate (νC=C stretching vibration mode) under simultaneous exposure to UV light. A conversion (*x*) *versus* time curve was then monitored by following the evolution of the νC=C band area at different times (*A*_t_), *A*_t=0 _being the area of this band before starting the photopolymerization.


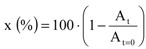


The slope of the conversion curve corresponds to the ratio of the polymerization rate (Rp) to the monomer concentration [M0]: d*x*/d*t* = Rp/[M0]. Its maximum value was used to compare the reactivity of formulations with or without nanofiller mixture.

All the irradiation experiments were performed at ambient temperature in the presence of air. The obtained nanocomposites were respectively called CS_1_ and CS_2_ nanocomposite.

### 3.3. Characterization

Nanofillers were characterized by powder X-ray diffraction (XRD) on a Philips X-pert diffractometer operating with Cu-Kα radiation (λ = 0.15418 nm), between 3° and 70° 2θ with a step size of 0.02° per 2 s.

Elemental analyses of Fe and Na were performed by X-ray fluorescence (XRF) with a Magix Philips (2.4 kW) apparatus. The samples were packed into pellets. Prior to analysis, calibration was performed with mixtures of SiO_2_ and MgCl_2_ at different concentrations. 

Phase transformations of different iron oxides (hematite, goethite, maghemite) were determined using a thermogravimetric analyzer with a TGA/DTA 851e apparatus from Mettler-Toledo Instruments in the temperature range of 25 to 800 °C with a scanning rate of 5 °C/min under air.

Transmission electron microscopy (TEM) images of the nanofillers were taken by placing a drop of the sample dispersed in chloroform onto a carbon film supported by a copper grid. A Philips EM 120 electron microscope operating at 120 kV was used. The nanocomposites obtained after UV curing were cut by means of a microtome (LKB model 8800) after cooling of the sample to 77 K and placed on the observation grid to obtain TEM images.

The influence of the nanofillers on the optical properties (transparency and gloss) of UV-cured samples was determined by means of a UV–visible spectrophotometer (Beckman DU 7400; Villepinte, France) and by a 20° gloss-meter (micro-TRI-gloss from BYK Gardner), respectively. Gloss measurements quantify how shiny a coating is and how the presence of fillers could affect it. They measure the amount of light reflected at the specular angle which is equal but opposite to the angle of incidence. Standard test method for specular gloss generally specifies at which angle gloss is measured. In the present study, gloss was measured at 20°.

The viscoelastic properties of the samples were determined by dynamic mechanical analysis (DMA-Q 800 from TA-Instruments) operating at 1 Hz frequency and 20 µm amplitude, with a 5°/min temperature rising rate. For these experiments, 100 µm films were cured using an industrial-type UV-line (Minicure (IST) UV Conveyor System), which was operated at belt speeds between 5 and 60 m/min, *i.e.*, UV doses of 950 and 50 mJ/cm^2^ per pass, respectively.

The pendulum hardness of polymer film (with or without nanofillers) was determined by using “Elcometer Model 3030 Pendulum hardness tester” on 100 µm thick films cured with the UV conveyor system. The “pendulum hardness” of coatings is reported in terms of damping time (in seconds) of the pendulum rested on the coating surface for a swing amplitude of könig pendulum going from 6° to 3°.

## 4. Conclusions

An original method was used to prepare new nanofillers by generating maghemite nanoparticles (γFe_2_O_3_) in the interlayer space of montmorillonite (MMT). By this process, up to 2.5 wt.% of iron was incorporated leading to a partial exfoliation of the montmorillonite. The γFe_2_O_3_-MMT/polymer nanocomposites loaded with 1 or 2 wt.% of nanofillers were then successfully obtained by photopolymerization of difunctional acrylate monomers. This offers advantages of UV-curing technology, namely, a high speed hardening of a solvent-free resin at room temperature. No inhibition effect was observed due to the presence of the nanofillers. An interesting improvement of the storage modulus of the composites was obtained for 2 wt.% of nanofillers compared to the crude polymer. Studies on the magnetic properties of these nanocomposites are under progress.

## References

[1] Rong M.Z., Zhang M.Q., Zheng Y.X., Zeng H.M. (2001). Improvement of tensile properties of nano-SiO2/PP composites in relation to percolation mechanism. Polymer.

[2] Li Y., Yu J., Guo Z. (2003). The influence of interphase on nylon-6/nano-SiO2 composite materials obtained from *in situ* polymerization. Polym. Int..

[3] Novakova A., Smirnovb E.V., Gendler T.S. (2006). Magnetic anisotropy in Fe3O4-PVA nanocomposites as a result of Fe3O4-nanoparticles chains formation. J. Magn. Magn. Mater..

[4] Ali-zade R.A. (2004). Structure and magnetic properties of polymer microspheres filled with magnetite nanoparticles. Inorg. Mater..

[5] Dumont M.J., Reyna-Valencia A., Emond J.P., Bousmina M. (2007). Barrier properties of polypropylene/organoclaynanocomposites. J. Appl. Polym. Sci..

[6] Zhu J., He Q., Luo Z., Khasanov A., Li Y., Sun L., Wang Q., Wei S., Guo Z. (2012). Property manipulated polypropylene–iron nanocomposites with maleic anhydride polypropylene. J. Mater. Chem..

[7] Gass J., Poddar P., Almand J., Srinath S., Srikanth H. (2006). Superparamagnetic polymer nanocomposites with uniform Fe_3_O_4_ nanoparticle dispersions. Adv. Funct. Mater..

[8] Qiu G., Wang Q., Nie M. (2006). Polyaniline/Fe_3_O_4_ magnetic nanocomposite prepared by ultrasonic irradiation. J. Appl. Polym. Sci..

[9] Schmidt A.M. (2006). Electromagnetic activation of shape memory polymer networks containing magnetic nanoparticles. Macromol. Rapid Commun..

[10] He Q., Yuan T., Zhu J., Luo Z., Haldolaarachchige N., Sun L., Khasanov A., Li Y., Young D.P., Wei S. (2012). Magnetic high density polyethylene nanocomposites reinforced with *in situ* synthesized Fe@FeO core-shell nanoparticles. Polymer.

[11] Alexandre M., Dubois P. (2000). Polymer-layered silicate nanocomposites: Preparation, properties and uses of a new class of materials. Mater. Sci. Eng. Rev..

[12] Zhu J., Wei S., Haldolaarachchige N., Young D.P., Guo Z. (2011). Electromagnetic field shielding polyurethane nanocomposites reinforced with core–shell Fe–Silica nanoparticles. J. Phys. Chem. C.

[13] Roco M.C. (1999). Nanoparticles and nanotechnology research. J. Nanopart. Res..

[14] Kronmüller H., Fischer R., Bachmann M., Leineweber T. (1999). Magnetization processes in small particles and nanocrystalline materials. J. Magn. Magn. Mater..

[15] Barbic M. (2002). Single domain magnets in bio-medical applications. Eur. J. Cells Mater..

[16] Ciobanu C.C., Iconaru S.L., Gyorgy E., Radu M., Costache M., Dinischiotu A., Le Coustumer P., Lafdi K., Predoi D. (2012). Biomedical properties and preparation of iron oxide-dextran nanostructure by MAPLE technique. Chem. Cent. J..

[17] Neamtu J., Verga N. (2011). Magnetic nanoparticles for magneto-resonance imaging and targeted drug delivery. Dig. J. Nanomater. Biostruct..

[18] Mahoudi M., Simchi A., Imani M., Hafali U.O. (2009). Superparamagnetic iron oxide nanoparticles with rigid cross-linked polyethylene glycol fumarate coating for application in imaging and drug delivery. J. Phys. Chem. C.

[19] Massart R. (1980). Préparation de ferrofluides aqueux en l’absence de surfactant, comportement en fonction du pH et de la nature des ions présents en solution. C. R. Acad. Sci. Paris.

[20] Meneghetti P., Qutubuddin S. (2006). Synthesis, thermal properties and applications of polymer-clay nanocomposites. Thermochim. Acta.

[21] Betega de Paiva L., Morales A.R., Valenzuela Diaz F.R. (2008). Organoclays: Properties, preparation and applications. Appl. Clay Sci..

[22] Jaber M., Miehe-Brendle J. , Valtchev V., Mintova S., Tsapatsis M. (2008). Organoclays: Preparation, Properties and Applications. Ordered Porous Solids.

[23] Godowsky D.Y., Varfolomeev A.V., Efremova G.D., Cherepanov V.M., Kapustin G.A., Volkov A.V., Moskvina M.A. (1999). Magnetic properties of polyvinyl alcohol-based composites containing iron oxide nanoparticles. Adv. Mater. Opt. Electron..

[24] Zhitomirsky I., Niewczas M., Petric A. (2003). Electrodeposition of hybrid organic-inorganic films containing iron oxide. Mater. Lett..

[25] Yrkov G.Y., Gubin S.P., Pankratov D.A., Koksharov Y.A., Kozinkin A.V., Spichkin Y.I., Nedoseikina T.I., Pirog I.V., Vlasenko V.G. (2002). Iron (III) oxide nanoparticles in a polyethylene matrix. Inorg. Mater..

[26] Wilson J.L., Poddar P., Frey N.A., Srikanth H., Mohomed K., Harmon J.P., Kotha S., Wachsmuth J. (2004). Synthesis and magnetic properties of polymer nanocomposites with embedded iron nanoparticles. J. Appl. Phys..

[27] Peeterbroeck S., Alexandre M., Dubois P., Thomas S., Valsaraj S.V., Meera A.P., Zaikov G. (2010). Processing of Polymer Nanocomposites: New Developments and Challenges. Recent Advances in Polymer Nanocomposites: Synthesis and Characterisation.

[28] Zhang Y., Evans J.R.G. (2012). Approaches to the manufacture of layered nanocomposites. Appl. Surf. Sci..

[29] Bitinis N., Hernandez M., Verdejo R., Kenny J.M., Lopez-Manchado M.A. (2011). Recent advances in clay/polymer nanocomposites. Adv. Mater..

[30] Akbari A., Talebanfard S., Hassan A. (2010). The effect of the structure of clay and clay modifier on polystyrene-clay nanocomposite morphology: A review. Polym.-Plast. Technol. Eng..

[31] Flesch C., Unterfinger Y., Bourgeat-Lami E., Duguet E., Delaite C., Dumas P. (2005). Poly(ethylene glycol) surface coated magnetic particles. Macromol. Rapid Commun..

[32] Azadmajiri J., Hojati-Talemi P., Simon G.P., Suzuki K., Selomulya C. (2011). Synthesis and electromagnetic interference shielding properties of iron oxide/polypyrrolenanocomposites. Polym. Eng. Sci..

[33] Agarwal T., Gupta K.A., Alam S., Zaidi M.G.H. (2012). Fabrication and characterization of iron oxide filled polyvinylpyrrolidone nanocomposites. Int. J. Compos. Mater..

[34] Laachachi A., Leroy E., Cochez M., Ferriol M., Cuesta J.M.L. (2005). Use of oxide nanoparticles and organoclays to improve thermal stability and fire retardancy of poly(methyl methacrylate). Polym. Degrad. Stab..

[35] Mamedov A., Ostrander J., Aliev F., Kotov N.A. (2000). Stratified assemblies of magnetite nanoparticles and montmorillonite prepared by the layer-by-layer assembly. Langmuir.

[36] Vassilios T., Georgi B., Vassilios T., Georgia B., Aristides B., Costas G., Hadjipanayis H., Mao D., Niarchos G., Hadjipanayis C. (2010). Immobilization of magnetic iron oxide nanoparticles on laponite discs—An easy way to biocompatible ferrofluids and ferrogels. J. Mater. Chem..

[37] Sugimoto T., Muramatsu A., Sakata K., Shindo D. (1993). Characterization of hematite particles of different shapes. J. Colloid Interface Sci..

[38] Bailey J.K., Brinker C.J., Mercartney M.L. (1993). Growth mechanisms of iron oxide particles of differing morphologies from the forced hydrolysis of ferric chloride solutions. J. Colloid Interface Sci..

[39] Fang J., Kumbhar A., Zhou W.L., Stokes K.L. (2003). Nanoneedles of maghemite iron oxide prepared from a wet chemical route. Mater. Res. Bull..

[40] Cudennec Y., Lecerf A. (2005). Topotactic transformations of goethite and lepidocrocite into hematite and maghemite. Solid State Sci..

[41] Mazo-Zuluaga J., Barrero C.A., Diaz-Teran J., Jerez A. (2003). Thermally induced magnetite–haematite transformation. Hyperfine Interact..

[42] Keller L., Decker C., Zahouily K., Benfarhi S., Meins J.M.L., Miehe-Brendle J. (2004). Synthesis of polymer nanocomposites by UV-curing of organoclay–acrylic resins. Polymer.

[43] Decker C., Keller L., Zahouily K., Benfarhi S. (2005). Synthesis of nanocomposite polymers by UV-radiation curing. Polymer.

[44] Reinholdt M., Miehe-Brendle J., Delmotte L., Tuilier M.-H., le Dred R., Cortes R., Flank A.-M. (2001). Fluorine route synthesis of montmorillonites containing Mg or Zn and characterization by XRD, thermal analysis, MAS NMR, and EXAFS spectroscopy. Eur. J. Inorg. Chem..

[45] Reinholdt M., Miehe-Brendle J., Delmotte L., le Dred R., Tuilier M.-H. (2005). Synthesis and characterization of montmorillonite-type phyllosilicates in a fluoride medium. Clay Miner..

